# A Microfluidic-Based Multi-Shear Device for Investigating the Effects of Low Fluid-Induced Stresses on Osteoblasts

**DOI:** 10.1371/journal.pone.0089966

**Published:** 2014-02-27

**Authors:** Weiliang Yu, Hong Qu, Guoqing Hu, Qian Zhang, Kui Song, Haijie Guan, Tingjiao Liu, Jianhua Qin

**Affiliations:** 1 Section of Oral Pathology, College of Stomatology, Dalian Medical University, Dalian, China; 2 Section of Orthodontics, College of Stomatology, Dalian Medical University, Dalian, China; 3 Department of Biotechnology, Dalian Institute of Chemical Physics, Chinese Academy of Sciences, Dalian, China; 4 State Key Laboratory of Nonlinear Mechanics (LNM), Institute of Mechanics, Chinese Academy of Sciences, Beijing, China; University of California, San Diego, United States of America

## Abstract

Interstitial fluid flow (IFF) within the extracellular matrix (ECM) produces low magnitude shear stresses on cells. Fluid flow-induced stress (FSS) plays an important role during tissue morphogenesis. To investigate the effect of low FSS generated by IFF on cells, we developed a microfluidic-based cell culture device that can generate multiple low shear stresses. By changing the length and width of the flow-in channels, different continuous low level shear stresses could be generated in individual cell culture chambers. Numerical calculations demonstrate uniform shear stress distributions of the major cell culture area of each chamber. This calculation is further confirmed by the wall shear stress curves. The effects of low FSS on MC3T3-E1 proliferation and differentiation were studied using this device. It was found that FSS ranging from 1.5 to 52.6 µPa promoted MC3T3-E1 proliferation and differentiation, but FSS over 412 µPa inhibited the proliferation and differentiation of MC3T3-E1 cells. FSS ranging from 1.5 to 52.6 µPa also increased the expression of Runx2, a key transcription factor regulating osteoblast differentiation. It is suggested that Runx2 might be an important regulator in low FSS-induced MC3T3-E1 differentiation. This device allows for detailed study of the effect of low FSS on the behaviors of cells; thus, it would be a useful tool for analysis of the effects of IFF-induced shear stresses on cells.

## Introduction

Interstitial fluid, flowing within the interstitium of the extracellular matrix (ECM), transports nutrients and signaling molecules between blood vessels, lymphatic capillaries, and ECM. Unlike blood flow, the velocity of interstitial fluid flow (IFF) is relatively slow, reaching microns per second [1], [2]. Besides its role in mass transport, IFF provides a specific mechanical environment for the cells, which is significant to cells’ physiological and pathological activities [3]–[8]. Low shear stress (µPa) generated by IFF plays an important role in the cellular mechanotransduction, by which cells transduce mechanical stimuli into a biochemical response.

Bone is composed of a collagen-hydroxyapatite matrix that forms a complex network of lacunae-canaliculi channels containing osteocytes and osteoblasts. Interstitial fluid fills in the microscopic network of lacunae and canaliculi. Mechanical loading and bending of bones can move the interstitial fluid, which applies shear stresses on osteoblasts and osteocytes. Fluid flow-induced shear stress (FSS) on these cells has been investigated extensively to assess its effects on cell differentiation, proliferation and function, by subjecting mesenchymal stem cells, osteoblasts and osteocytes to fluid shear stress [8]–[16]. Hayward et al. found that interstitial fluid velocity and tissue shear stress are key mechanical stimuli for the differentiation of skeletal tissues. Another *in vivo* study demonstrated that increased IFF inhibited bone loss in hindlimb-suspended mice [15]. Studies *in vitro* also showed that primary and clonal osteoblast-like cells responded to shear stress with increased cell proliferation and differentiation [11], [12]. Furthermore, Delaine-Smith et al. reported that ALP activity, collagen production and calcium deposition were enhanced in human progenitor dermal fibroblasts by FSS when these cells were cultured in osteogenic media [17]. Mechanical regulation signaling pathways in bone include activation of kinases (Akt, MAPK, FAK), β-catenin, GTPase, and calcium signaling [18]. Regarding the signaling pathway regulating FSS-induced osteoblast differentiation, You et al. demonstrated that the expression of osteopontin, an important bone matrix protein, was regulated by FSS via intracellular calcium mobilization and activation of mitogen-activated protein kinase (MAPK) in MC3T3-E1 cells [19]. Arnsdorf et al. reported that oscillatory fluid flow regulates osteogenic differentiation via the activation of RhoA, ROCKII and Runx2 [20]. Runx2 is an essential transcription factor for osteoblast differentiation and bone formation; Runx2-deficient mice completely lack bone formation due to the arrest of osteoblast maturation [21], [22].

A parallel-plate fluid flow chamber is widely utilized to mimic the *in vivo* fluid flow-filled environment of osteoblasts and osteocytes. The chamber, in its original design, is capable of producing well-defined shear stress in the range of 0.001–3 Pa. Shear stress is generated by flowing fluid through the chamber over the immobilized substrate under controlled kinematic conditions using a syringe pump. The advantages of the parallel-plate flow chamber include generating a constant shear stress on cells over a defined time period and its simple operation. A prominent disadvantage of this device is that only a single velocity can be generated in one experiment. In addition, it is difficult to generate low magnitude shear stresses (µPa) using the parallel-plate chamber. Complex bioreactors have been designed that allow different stress mimics such as compressive, shear and rotational forces to be applied to three-dimensional cultured cells [23]. However, the flow distribution generated by these instruments was non-uniform and FSS could not be controlled precisely. To overcome these limitations, a simple and controllable fluid flow *in vitro* model is required.

Recent technological advances have shown great progress in the biological field. Microfabricated devices have been developed to facilitate both applied and basic research concerning the biology of cells and tissues. As a new technology, microfluidics has aroused increasing interest in the biological and medical sciences for requiring less time, reduced sample consumption and low cost [24], [25]. A distinct advantage of microfluidic technology is the precise control and manipulation of fluids that are geometrically constrained to a sub-millimeter scale. Furthermore, the power of microfluidics for large-scale and high-throughput screening has been demonstrated. Thus, microfluidic technology is an ideal tool to study the effects of fluid flow on cells. Various studies based on microfluidic technology have been carried out to investigate FSS effects on the behaviors of osteoblasts, endothelial cells, and fibroblasts [26]–[34]. Most of these devices, however, can only generate one shear stress on cells cultured on the device in a single experiment. Kou et al. reported a multi-shear microfluidic system to study FSS-induced cytosolic calcium concentration dynamics of osteoblasts [26]. This device can offer controllable multi-shear stresses from 0.03 to 0.3 Pa and perform quantitative comparisons of shear stress-induced changes of calcium intensity in osteoblasts. However, the authors did not report the generation of low magnitude FSS (µPa) by this device. The effect of IFF-induced low FSS on osteoblasts is still unclear.

To investigate the effect of low FSS generated by IFF on the behaviors of osteoblasts, we developed a microfluidic system that generates four types of FSS from 1.5 to 412 µPa on a single device. FSS-induced proliferation and differentiation of MC3T3-E1 was studied using this device. Our results suggest that adequate FSS could promote MC3T3-E1 proliferation and differentiation, but over-loading of FSS inhibited proliferation and differentiation of pro-osteoblastic cells.

## Materials and Methods

### Fabrication of the Microfluidic Device

We constructed a device composed of two layers: the Polydimethylsiloxane (PDMS) layer and glass substrate. The PDMS layer was fabricated by replicate molding the master, which was prepared by spin coating SU8-2035 negative photoresist (Microchem Corp., US) onto a glass wafer, and then patterned by photolithography. Sylgard 184 PDMS base and curing agent (Sylgard Silicone elastomer 184, Dow Corning Corp., US) were mixed thoroughly (10∶1 by mass), degassed under vacuum, and poured onto the master. The polymer curing process was conducted in an oven for 1 hr at 80°C. After cooling, the PDMS layer was gently peeled off of the master and trimmed to size. Inlet and outlet holes were created by punching through the PDMS with a razor-sharp punch. The piece of PDMS was bonded to a glass slide irreversibly after oxygen plasma treatment for 60 s. Prior to use, the device was sterilized with UV light for 30 min.

### Cell Culture in the Microfluidic Device

**Figure 1:**
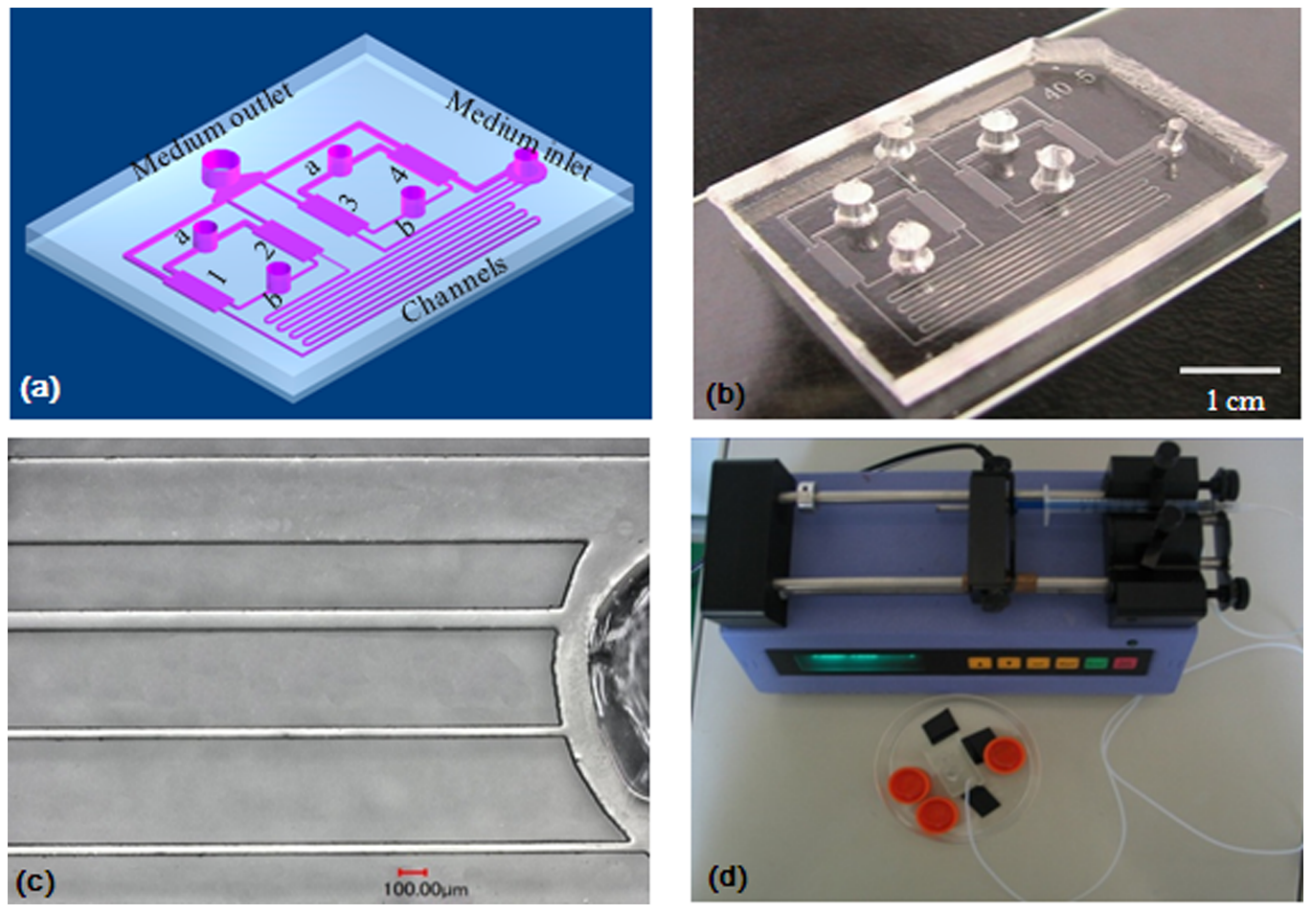
Chip design. (a) Schematic representation of the microfluidic chip, including four cell culture chambers connected to channels. (b) Chip photo. (c) The diameter of each channel. (d) The chip was connected with a programmable syringe pump, which provides precise control of the flow rate at 0.08 µl/min over 48 h. Scale bar = 100 µm.

MC3T3-E1, an osteoprogenitor cell line from mouse, was purchased from China Center for Type Culture Collection. It was cultured at 37°C with 5% CO_2_ and 95% relative humidity in α-MEM (Sigma, US) supplemented with 10% fetal bovine serum (Hyclone, US), 100 U/ml penicillin, and 100 U/ml streptomycin. When the cells grew to 70% confluency, they were detached from flask by 0.25% trysin-EDTA. Then the cells were centifuged and suspended at 10^6^ cells/ml in fresh medium. As shown in Fig. 1a, there are four cell culture chambers in the microfluidic device. The suspended cells were seeded in the cell culture chambers via cell inlets. Then the device was placed inside a 37°C incubator with 5% CO_2_ and 95% relative humidity. After 24 hr, MC3T3-E1 cells attached to the bottom of cell culture chambers. Then a syringe pump was connected with the microfluidic device via medium inlet to perform continuous perfusion culture for 24 hrs.

### Shear Stress Modeling

**Figure 2:**
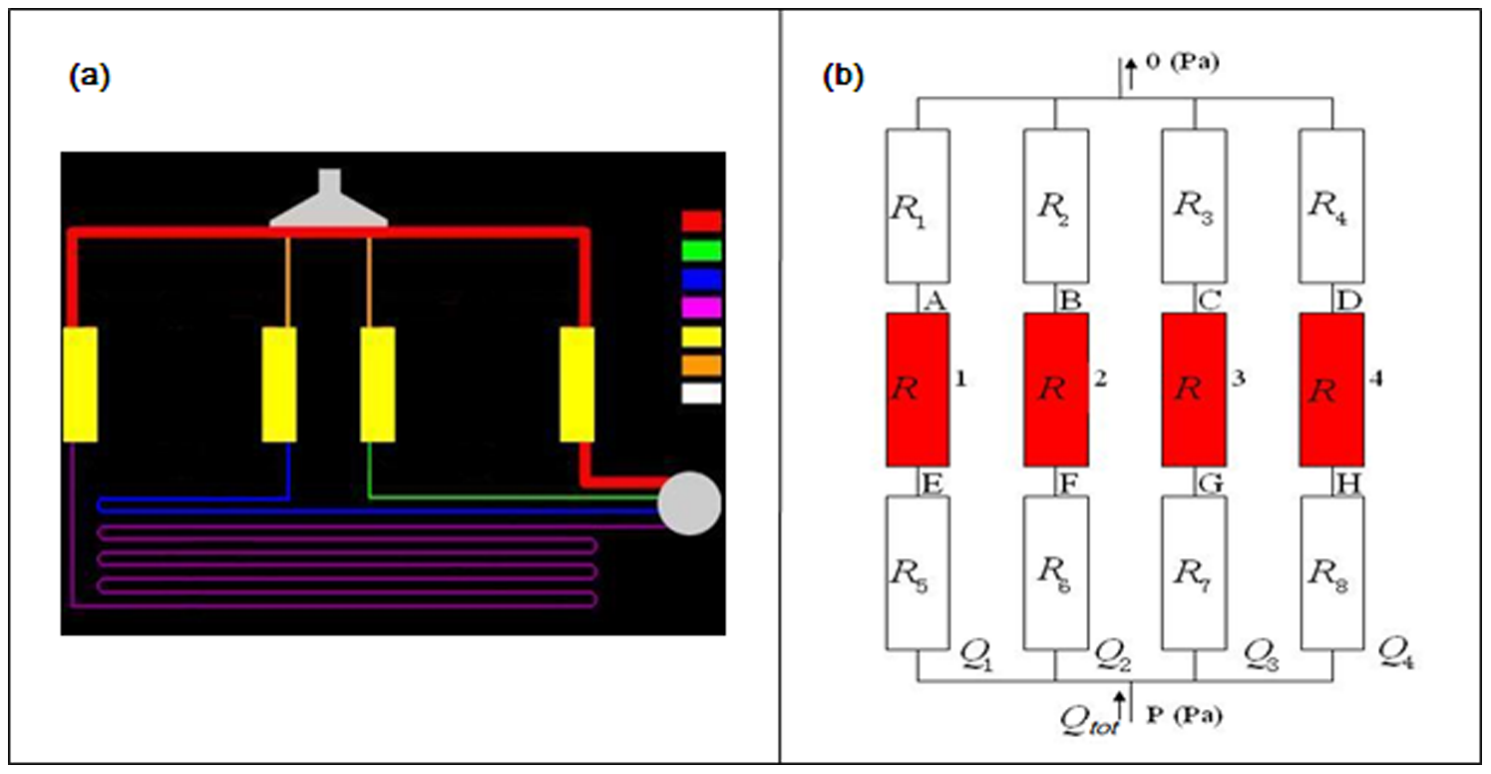
Numerical modeling. (a) Simplified microfluidic network. (b) The equivalent circuit diagram corresponding to the microfluidic network of (a), where *Ri* is the hydraulic resistance of the microchannel, and *R* is the hydraulic resistance of the identical culture chamber.

It is important to understand and control the FSS on cell surface for cell culture. In contrast to a straight microchannel in which the Poiseuille flow model can be used to estimate the wall shear stress, the present complex microfluidic network has to be simplified first and then simulated using the Computational Fluid Dynamics (CFD) method. As can be seen from Fig. 1a–b, the culture chip consists of multiple long and small microchannels and relatively larger cell culture chambers. It is computationally intensive to directly simulate the flow field in the whole microfluidic systems because the computational domain is three dimensional (3D) and the length scales differ significantly. Meanwhile, only the shear stresses within the four cell culture chambers are important to our investigation. The 3D flow field in the chamber can be obtained by CFD simulation if the pressure values at the inlet/outlet are known. Taking advantage of the Stokes flow (Re <1) property in the present cell culture chips, we are able to apply the well-know methods from the electric circuits theory to the microfluidic networks. Analogous to an electrical circuit, individual channel sections can be treated as resistances within the flow circuit [35]. Since the introduction channels were blocked after cells were introduced to the chambers, the introduction channels can be excluded and the microfluidic network in Fig. 1a can be further simplified to that shown in Fig. 2a. The equivalent electric circuit corresponding to the simplified fluidic system was shown in Fig. 2b, where *R_i_* is the hydraulic resistance of the each microchannel connected to the chambers and *R* is the hydraulic resistance of the identical culture chamber.

The hydraulic resistance of the 3D rectangular channel can be derived via Fourier series expansions. To avoid the computational cost in solving Fourier series expansions, however, we use an approximate version in algebraic form,
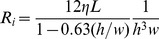
(1)where 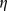 is the liquid viscosity, *L* the channel length, *h* and *w* (always *h<w*) the channel height and width, respectively. The additive law for the inverse resistances links the total hydraulic resistance *R_tot_* to the resistances of the individual channels in the flow circuit in Fig. 2b,



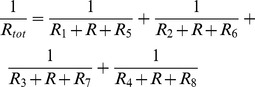
(2)The pressure drop Δ*p* over this flow circuit then can be calculated for a constant flow rate *Q_tot_*,
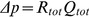
(3)


After simple algebraic manipulating, we can also obtain the pressure values at the inlets and outlets of the individual culture chambers: *p_E_, p_F_, p_G_, p_A_, p_B_, p_C_, p_D_*, just like obtaining the voltage drops in a given electric circuit. These pressure values are then used as inlet and outlet pressure conditions to simulate the 3D flow field and thus flow rate in each culture chamber using CFD method. The incompressible Navier-Stokes equations are used to model the steady flow field in the culture chambers. It worth noting that in the present simulations we do not consider the effect of the cells on the flows. The computational domain is discretized by about 26,000 hexahedral meshes and solved by finite volume method, along with the aforementioned inlet/outlet pressure conditions and no-slip boundary conditions at the chamber walls. Both velocities the chamber and shear stress at the bottom boundaries of the domain are collected.

### Proliferation Index Calculation

The total number of cell in each culture chamber was calculated under microscope before perfusion culture. After perfusion culture, the cells were stained by Rhodamine 123 (Rh-123, Sigma, US),Hoechst 33342 (Molecular Probes, US) and propidium iodide (PI, Molecular Probes, US). Briefly, we introduced 2 µg/ml Rh-123 anf 4 µg/ml Hoechst 33342 into the culture chamber and incubated at 37°C for 40 min; rinsed with PBS; then labeled with 2 µg/ml PI at 37°C for 5 min and imaged under a fluorescent microscope (Olympus IX71, Japan). Then the total number of cells in each chamber was calculated. The increased cell number in each chamber:
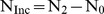
where “N_Inc_” represents the increased cell number. Subscripts denote either the initial cell number (N_0_), or the number at day 2 (N_2_). The proliferation index (P) in each cell culture chamber was defined as:
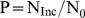



### Total RNA Isolation and RT-PCR

The total RNA was extracted from the cultured cells in each cell culture chamber for RT-PCR analysis using Total RNA Isolation Kit (Macherey-Nagel, Germany). Aliquots of 400 ng total RNA were reverse transcribed to cDNA using the PrimeScript™ 1st Strand cDNA Synthesis Kit for RT-PCR (Takara Biotechnology, Dalian, China). The resulting cDNA products were subjected to PCR amplification using the gene-specific primers for alkaline phosphatase (ALP), osteocalcin, Col I, Runx2, and GAPDH. The amplified products were electrophoresed in 2% agarose gel containing ethidium bromide with DL 2000 DNA Marker (Takara Biotechnology, Dalian, China). The specific primers used are listed in Table 1.

**Table 1:** Primers used for RT-PCR.

	Forward primer	Reverse primer
ALP	5′-TGAGCGACACGGACAAGA-3′	5′-GGCCTGGTAGTTGTTGTGAG-3
Osteocalcin	5′-CCAAGCAGGAGGGCAATA-3′	5′-AGGGCAGCACAGGTCCTAA-3′
Col I	5′-TCCCTTGGACATTGGTGCC-3′	5′-GTTCGTCTGTTTCCAGGGT-3′
Runx2	5′-CCGCACGACAACCGCACCAT-3′	5′-CGCTCCGGCCCACAAATCTC-3′
GAPDH	5′-TGACGGGAAGCTCACTGG-3′	5′-TCCACCACCCTGTTGCTGTA-3′

### Statistical Analysis

Statistical analyses were performed using SPSS version 13.0 for Windows. Student *t* test was used to confirm comparisons of binary variables. Each experiment was conducted at least three times. Significance was identified as *p* value of less than 0.05.

## Results and Discussion

### Chip Design

The microfluidic device developed in this study comprises four cell culture chambers connected with individual flow-in and flow-out channels (Fig. 1a, b). The four chambers are identical in size, 4265 µm in length and 1265 µm in width. Chambers 1 and 2 share the same cell inlet and outlet. Chambers 3 and 4 share the same cell inlet and outlet. Four flow-in channels share one medium inlet, while four flow-out channels share one medium outlet. The widths of flow-in channels 1, 2, 3, and 4 are 50 µm, 50 µm, 90 µm, and 400 µm, respectively (Fig. 1c). The lengths of flow-in channels 1, 2, 3, and 4 are 1350 mm, 30 mm, 13 mm, and 4.5 mm, respectively. The different widths and lengths of the flow-in channels are used to generate different FSS in the bottom of the four cell culture chambers. By contrast, the differences in widths and lengths of the flow-out channels have little effect on changes in the shear stress in the four cell culture chambers. The cell culture chambers and channels have the same height of 100 µm. To generate continuous FSS on the bottom of cell culture chambers, the device was connected with a syringe pump via the medium inlet to perform perfusion (Fig. 1d).

A widely used *in vitro* model to study shear stresses on cells in the biological field is the parallel chamber [36], [37]. However, because only a single shear stress can be generated in one experimental condition using the parallel chamber, this device is not an ideal platform to study the effect of different shear stresses on cells in the same experiment. It would be convenient to generate different shear stresses on a single microfluidic device. So, the effects of different shear stresses on cells could be compared in a single experiment. One study described a multi-shear microfluidic device for quantitative analysis of calcium dynamics. Different shear stresses were produced in the different-sized cell culture chambers [26]. However, because the number of cells in each chamber is different, it is difficult to quantify the exact effect of FSS on cells. A prominent advantage of our microfluidic device is to generate different shear stresses by changing the width and length of flow-in channels that connect the four cell culture chambers. The four cell culture chambers are identical in size, thus there are likely the same number of cells in each chamber. The effect of different shear stresses on the same number of cells can be studied easily using the device.

### Numerical Results

**Figure 3:**
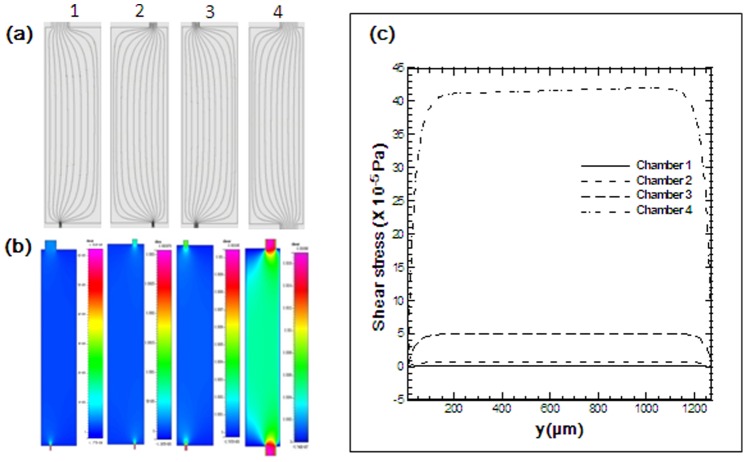
Shear stress calculations in four chambers. (a) Flow streamlines at the middle height plane. (b) Shear stress at the bottom wall. (c) Distributions of the wall shear stress in the y-direction.

To mimic IFF, different perfusion flow rates of 0.06 µl/min, 0.08 µl/min, 0.1 µl/min, and 0.15 µl/min are applied to generate different shear stresses in the four cell culture chambers. Because the velocities and shear stresses are linearly proportional to the flow rates in the Stokes flows, we only need to simulate one flow rate. The cell culture media is assumed to be identical to pure water with a density of 1000 *kg/m^3^* and viscosity of 0.001 *kg/m/s*. Four chambers have identical length *l_x = _*4264.6 µm, width *l_y_* = 1264.6 µm, and height *l_z_* = 100 µm. The culture chambers are connected to the flow-in channels and flow-out channels at different positions, which is also taken into account in the CFD simulations. Fig. 2 plots the shear stress at the bottom wall (*z* = 0) and flow streamlines at the middle height plane (*z* = 50 µm) under the perfusion flow rate of 0.08 µl/min. The numerical calculations demonstrate uniform stress distribution in the bottom area of the chamber, except in the vicinity of the inlet, outlet, and side walls. This observation is further confirmed by the wall shear stress curves shown in Fig. 3. These curves are plotted in the y-direction at *x* = *l_x_*/2 = 2132.3 *µm*. Under a flow rate of 0.08 µl/min, the averaged flow velocities in chambers 1 to 4 are 0.04 µm/s, 0.19 µm/s, 1.33 µm/s, and 16 µm/s, respectively. The averaged bottom wall shear stresses of chambers 1 to 4 are estimated to be 1.5 µPa, 7.7 µPa, 52.6 µPa, and 412 µPa, respectively. The ratio of shear stresses of chambers 1 to 4 is approximately 1∶ 5∶ 35∶ 275.

Unlike vascular endothelial cells exposed to high FSS generated by blood flow, most of the cells *in vivo* are exposed to low magnitude FSS generated by interstitial fluid. To mimic the physiological condition of the flow velocity of interstitial fluid (microns per second), we tried different perfusion flow rates of 0.06 µl/min, 0.08 µl/min, 0.1 µl/min, and 0.15 µl/min. The average flow velocity ranged from 0.04 to 16 µm/s in chamber 1 to 4 under a flow rate of 0.08 µl/min, similar to conditions *in vivo*. Therefore, the device developed in our study is suitable to investigate the effect of interstitial fluid-induced low magnitude FSS on cells.

### Effect of Low IFF on the Proliferation of MC3T3-E1

**Figure 4:**
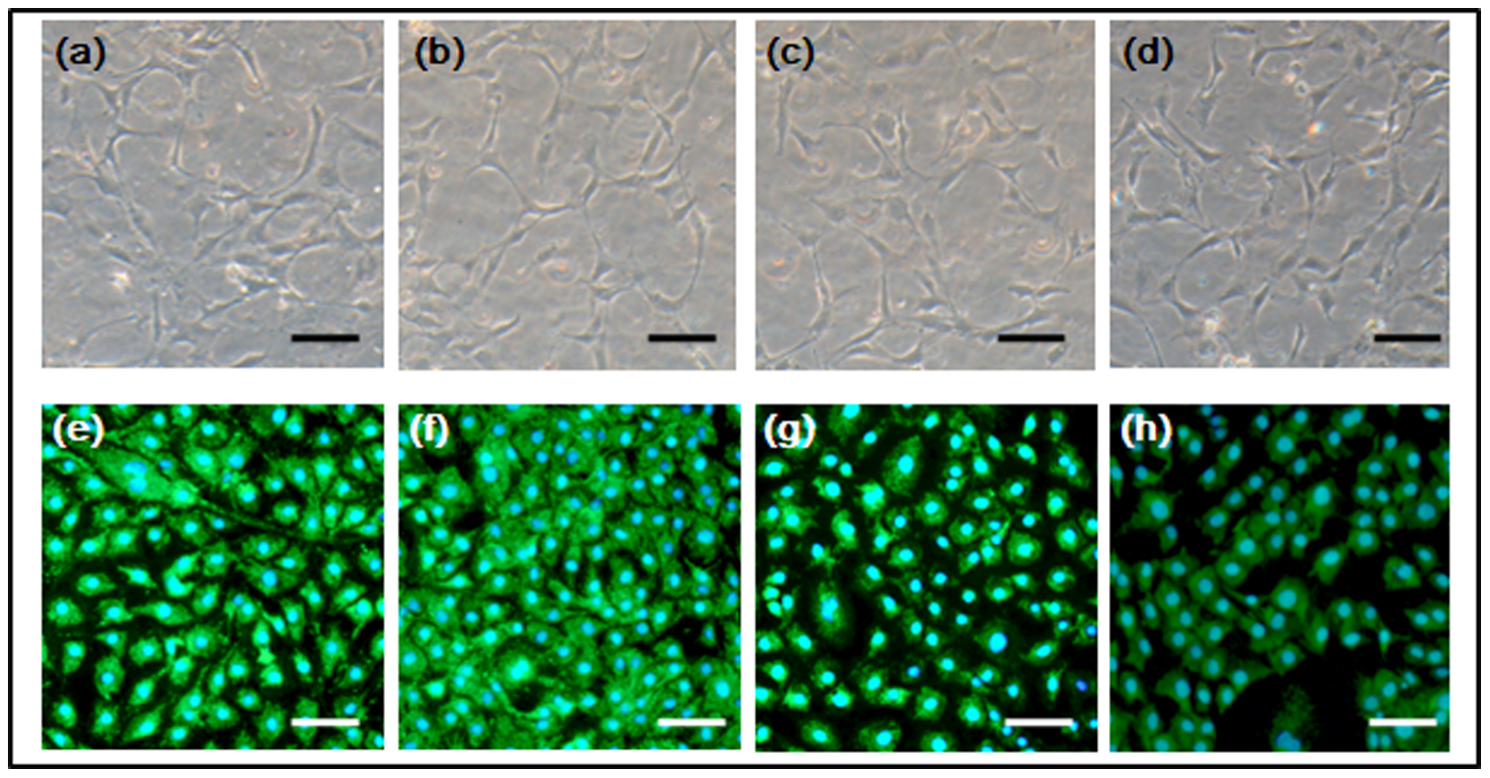
MC3T3-E1 cells in four chambers with perfusion culture for 48 h. (a–d) MC3T3-E1 cells in chambers 1, 2, 3, and 4, respectively, before perfusion culture (bright field). (e–h) MC3T3-E1 cells in chambers 1, 2, 3, and 4, respectively, with perfusion culture for 48 h, then stained by Rh123-Hoechst-PI. Scale bar = 100 µm.

**Figure 5:**
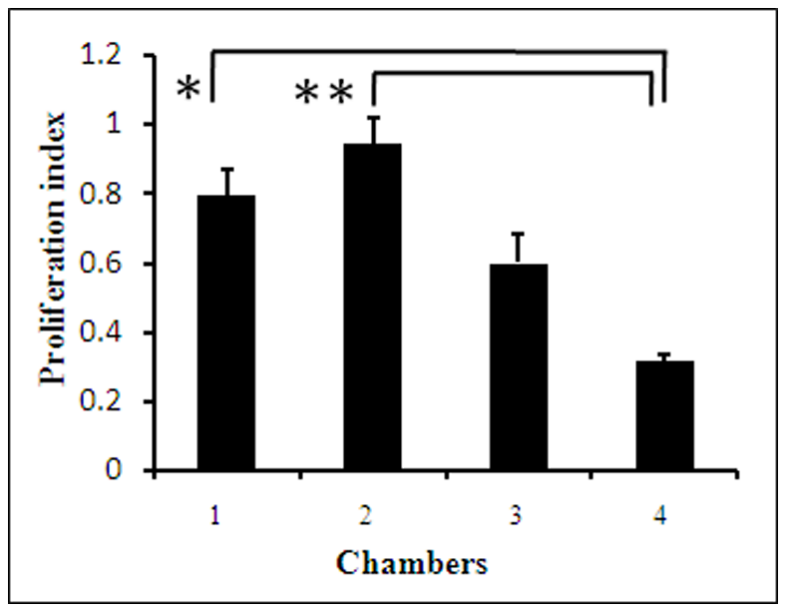
The proliferation index of MC3T3-E1 cells in chambers 1, 2, 3, and 4. The proliferation index of MC3T3-E1 cells in chamber 4 was lower, compared to cells in chambers 1 and 2. The single asterisk indicates *p*<0.05 and the couple asterisk indicates *p*<0.001.

To study the effect of low IFFs on the proliferation of osteoblasts, MC3T3-E1 cells were seeded in the cell culture chambers 1 to 4. When the cells attached to the bottom and reached over 70% confluence, a syringe pump was connected to the microfluidic device via medium inlet to perform continuous perfusion culture. Different perfusion flow rates of 0.06 µl/min, 0.08 µl/min, 0.1 µl/min, and 0.15 µl/min were applied. The flow rate of 0.08 µl/min was found to be suitable for the cells in the four chambers; some cells in chamber 4 were detached at the flow rate over 0.1 µl/min. After MC3T3-E1 cells were stimulated by FSS for 48 h, the cells in chamber 1 to 4 were stained by Rh-123, Hoechst 33342 and PI (Fig. 4). The cells in all the chambers presented as flat with protrusions. The cellular viability was very good. There were few, if any, PI positive cells (apoptotic cells). This might be due to the weak attachment of apoptotic cells, which were swept away by the flowing culture medium. Compared to the state before perfusion culture, the cell number in each chamber increased. The proliferation rates of cells in chambers 1, 2, 3, and 4 were assessed (Fig. 5). Compared to that in chambers 1 and 2, the cell proliferation rate was significantly lower in chamber 4. The cell proliferation rate also decreased in chamber 3, but not significantly. The cells in chamber 2 showed the highest proliferation, but no significant difference was observed between chambers 1 and 2.

Most of the previous *in vitro* experiments have investigated the effects of shear stresses of 0.01–10 Pa on osteoblast proliferation [12], [13], [28]. Small magnitude shear stresses as low as 1–63 µPa could stimulate osteoblastic cell proliferation when stress was applied intermittently for 24 h [11]. Similar to that study, our results proved that IFF ranging from 1.5 to 7.7µPa stimulated osteoblast proliferation greatly. Furthermore, our results proved that the stimulation effect decreased when IFF was greater than 412 µPa. We also grew control group of cells in the microfluidic device without FSS. The proliferation rate of control was found to be a little lower than that of cells in chamber 1 (data not shown). These results suggest that low IFF (1.5–7.7 µPa) has a greater stimulation effect on osteoblast proliferation than high IFF (over 412 µPa).

### Effect of IFF on the Differentiation of MC3T3-E1

**Figure 6:**
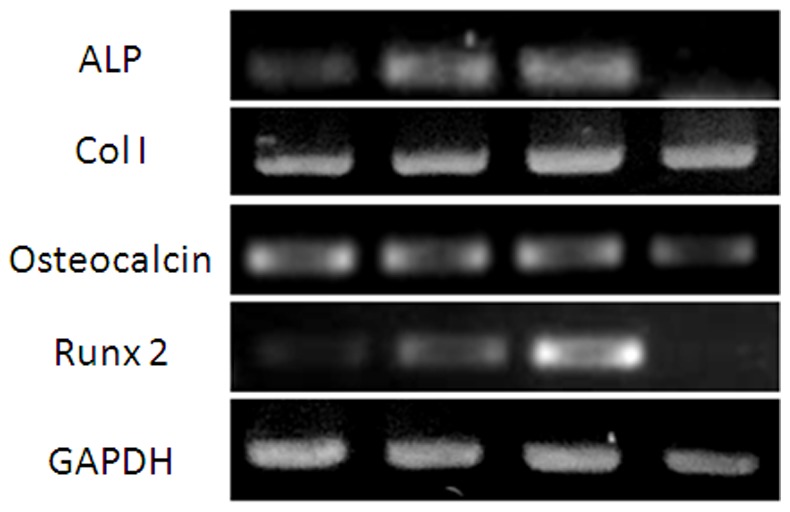
The differentiation of MC3T3-E1 cells in chambers 1, 2, 3, and 4. The expression of ALP, Col I, and osteocalcin increased gradually from chambers 1 to 3, then decreased in chamber 4. Runx2 expression showed a similar pattern to that of ALP and osteocalcin.

As demonstrated in our study showing that low FSS stimulated MC3T3-E1 proliferation, we wanted to know the effect of low FSS on osteoblast differentiation. After 48 h perfusion culture, the total RNA of MC3T3-E1 cells in the four culture chambers were isolated. The expression of several markers of osteoblast differentiation was analyzed by RT-PCR (Fig. 6). It was found that the expression of ALP, Col I, and osteocalcin increased gradually from chambers 1 to 3. There was negligible ALP expression of cells in chamber 4. Although Col I and osteocalcin expression were found in chamber 4, their levels were lower than those in chambers 1–3. This suggests that the differentiation of MC3T3-E1 cells increased from chambers 1 to 3, but decreased in chamber 4. To determine which transcription factor regulated the FSS-induced osteoblast differentiation, the expression of Runx2, the main transcription factor regulating osteoblast differentiation, was analyzed. It was found that the expression pattern of Runx2 in the four chambers was similar to that of ALP, Col I, and osteocalcin. It increased from chambers 1 to 3 gradually, but decreased in chamber 4. Almost no expression of osterix, another transcription factor regulating osteoblast differentiation, was found in the cells in the four chambers (data were not shown).

Because bone is a porous material, the deformation of bone tissue during physiological levels of mechanical load induces a cyclic movement of IFF. However, the cellular and molecular mechanisms by which stem cells respond to shear stress is not well known. It was reported that small magnitude shear stresses as low as 1–63 µPa stimulated ALP expression in primary human osteoblasts [11]. Another study reported that human bone marrow-derived mesenchymal stem cells showed a significant increase in ALP expression and a marked decrease in Col I when they were subjected to the laminar fluid flow of 1.2 Pa for 30 and 90 min [38]. However, no obvious change of Runx2 expression was detected. Another study reported that the long-term (10 days) application of a defined and uniform level of fluid flow (1.2 µPa) on human bone marrow stromal cells reduced the number of cell doublings [39]. The different results might be due to different cells used and experimental conditions. Our results proved that differentiation of MC3T3-E1 cells was promoted only by low FSS (1.5–52.6 µPa). The Runx2 signaling pathway is a key regulator in low FSS-induced MC3T3-E1 differentiation.

### Conclusions

In conclusion, we developed a multi-shear microfluidic device for studying the effects of low FSS on cells. Multiple shear stresses ranging from 1.5 to 412 µPa could be generated in a single device. Low FSS-induced MC3T3-E1 proliferation and differentiation was studied further. It was proved that FSS ranging from1.5 to 52.6 µPa promoted MC3T3-E1 proliferation and differentiation, but high FSS (over 412 µPa) inhibited proliferation and differentiation of the pro-osteoblastic cells. Capable of modeling low FSS, the device is applicable for analysis of the effects of interstitial fluid-induced shear stress on cells.
